# Phase I/II study of carfilzomib, bendamustine, and dexamethasone (CBD) in patients with newly diagnosed multiple myeloma

**DOI:** 10.1038/s41408-020-0278-5

**Published:** 2020-02-03

**Authors:** Siyang Leng, Divaya Bhutani, Shahzad Raza, Amer Assal, Samuel Pan, Jianhua Hu, Alexander Wei, Markus Mapara, Suzanne Lentzsch

**Affiliations:** 1 Division of Hematology/Oncology, Columbia University Irving Medical Center, 161 Fort Washington Ave, New York, NY 10032 USA; 2 Saint Luke’s Health System, 4321 Washington St Ste 4000, Kansas City, MO 64111 USA; 3 Department of Biostatistics, Columbia University Mailman School of Public Health, 622 West 168th St, New York, NY 10032 USA; 4 Department of Medicine, Columbia University Irving Medical Center, 161 Fort Washington Ave, New York, NY 10032 USA

**Keywords:** Chemotherapy, Phase I trials

Dear Editor,

Despite remarkable improvements in treatment and survival, multiple myeloma remains incurable, and deep responses to induction, ideally with minimal residual disease (MRD) negativity^1^, are much desired and key determinants of long-term survival. Though proteasome inhibitor (PI) and immunomodulatory drug (IMID) combinations are standard for newly diagnosed patients in the United States^2,3^, PIs can also be combined with alkylating agents, allowing IMIDs to be saved for use at relapse. Combinations, such as bortezomib–bendamustine–prednisone^4^, and bortezomib–cyclophosphamide–dexamethasone^5^, are safe and have impressive activity as frontline therapies.

Bendamustine interferes with DNA replication and induces inhibition of mitotic checkpoints, inefficient DNA repair, and initiation of p53-dependent DNA-damage stress responses^6^. It has synergistic activity with bortezomib^7^. Carfilzomib is a second-generation, irreversible PI, which may be superior in efficacy to bortezomib^8^, and has promising activity in first-line therapy^9^. We hypothesized that combining bendamustine with carfilzomib will yield a tolerable and effective induction regimen.

We conducted a single-arm, single-center, open-label, phase I/II study of carfilzomib, bendamustine, and dexamethasone (CBD) in patients with newly diagnosed myeloma. The primary endpoint was to determine the maximum tolerated dose (MTD), defined as the highest dose at which ≤20% of patients experience dose-limiting toxicity (DLT). Secondary endpoints were overall response rate (ORR), time to response, progression-free survival (PFS), overall survival (OS), toxicity, and stem cell yield.

Patients were ≥18 years old, met diagnostic criteria for myeloma^10^, and had measurable disease, Eastern Cooperative Oncology Group (ECOG) performance status 0–2, and adequate renal, cardiac, and hepatic function. Patients with plasma cell leukemia were excluded.

**Fig. 1:**
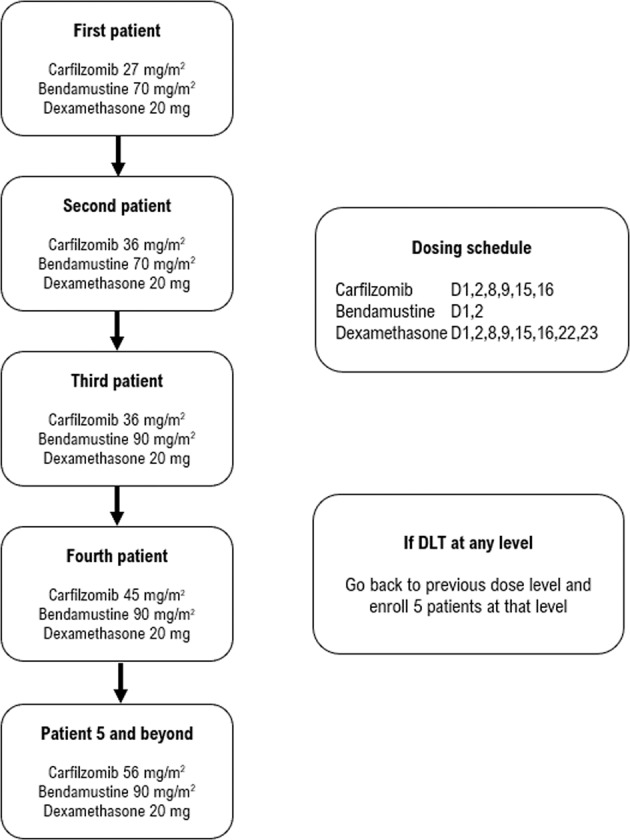
Trial schema for this phase I/II study assessing carfilzomib, bendamustine, and dexamethasone in newly diagnosed myeloma patients.

Administration of CBD is shown in Fig. 1. All agents were given intravenously. Carfilzomib was started at 20 mg/m^2^ (days 1, 2; cycle 1), after which it was escalated to the doses specified in Fig. 1. Each cycle was 28 days. Patients eligible for autologous stem cell transplant (SCT) received four cycles of CBD, underwent stem cell harvest (SCH), and then received four more cycles of CBD followed by SCT. SCT-ineligible patients received eight cycles of CBD. Whether SCT-eligible or ineligible, maintenance with carfilzomib 36 mg/m^2^ on days 1, 2, 15, and 16 for up to 2 years was recommended. However, the protocol was later amended to allow for investigator and patient discretion regarding maintenance, and other options including lenalidomide were allowed. Supportive care consisted of antiviral prophylaxis against herpes zoster, and zoledronic or pamidronic acid for treatment of myeloma bone disease.

We used a two-stage up-and-down dose escalation algorithm (Storer’s Design BD) to evaluate dosing^11^. Adverse events (AEs) were graded by using CTCAE, version 4.0^12^. An AE was considered a DLT if it occurred in cycle 1, was deemed related to study treatment, and was one of peripheral neuropathy ≥grade 2, any non-hematologic AE ≥ grade 3, neutropenia grade 4 lasting ≥7 days or with fever, thrombocytopenia grade 4 lasting ≥7 days or with bleeding, or any AE requiring a dose reduction during cycle 1 or a delay in the start of cycle 2.

Only patients who received ≥2 cycles of treatment were considered evaluable for response. Responses were determined by the treating physician according to the International Myeloma Working Group (IMWG) consensus criteria^13^. All complete responses (CR) were confirmed by bone marrow aspiration. MRD status was determined by flow cytometry, which conformed to the EuroFlow procedure, with a cutoff of 10^−5 ^^14^.

The trial targeted 34 patients for enrollment, based on the assumption that 5 patients would be enrolled on each of 6 dose levels, with an additional 4 patients included to make up for screen failures. Due to slow accrual and loss of funding, the trial was closed after 20 patients were enrolled. At this point, we evaluated the probability to observe a DLT by the Clopper–Pearson method. Survival was evaluated by Kaplan–Meier analysis. For all outcomes, data cutoff was March 1, 2019. Analyses were performed using RStudio Version 1.1.463 and SAS 9.4.

This trial was conducted in accordance with guidelines from the U.S. FDA, ICH GCP, and Declaration of Helsinki, and is registered at clinicaltrials.gov (NCT02002598). It was approved by our Institutional Review Board. All participants provided written informed consent.

Twenty patients with newly diagnosed myeloma were accrued. Median age was 65 (range 48–74); 14 (70%) were male; 7 (35%) were Hispanic. Three (15%) were R-ISS 3, and one (5%) had high-risk cytogenetics. All patients were evaluable for safety; 19 were evaluable for response.

Sixteen patients completed eight cycles of CBD. Four discontinued early for neutropenia grade 3 after five cycles, prolonged grade 2–3 thrombocytopenia after six cycles, prolonged grade 2 fatigue after seven cycles, and acute transaminitis grade 3 during cycle 1 (this last patient was found to be taking four supplements, including high-dose aloe vera; the transaminitis was attributed to supplement use, and did not recur after the patient was taken off study and treated with bortezomib–dexamethasone). Of the 19 patients receiving ≥2 cycles, 15 (79%) were eligible for SCT, 13 (68%) underwent SCT, and 2 declined. SCH was not impacted by CBD (data not shown). For maintenance, 10 of 19 (53%) received carfilzomib, 5 lenalidomide, 2 were lost to follow-up, and 2 declined. Of the ten patients receiving carfilzomib, median duration of use was 15 months. Median duration on study treatment was 9.7 months (range 6.5–36). None of the patients discontinued study treatment due to disease progression.

We did not observe any DLT. Our 95% confidence interval for observing one DLT at our highest dose level of 5 was 0–19.5%. Since this interval is <20%, we conclude that dose level 5 (carfilzomib 56 mg/m^2^, bendamustine 90 mg/m^2^, and dexamethasone 40 mg) can be established as the MTD, despite having recruited fewer patients than planned. The most common severe treatment-related AEs were hematologic. Grade 3/4 lymphocytopenia occurred in 90% of patients, neutropenia 40%, and thrombocytopenia 20%. Grade 3 anemia occurred in 20% (no grade 4). Notable non-hematologic AEs were grade 3/4 infection in 20% of patients (typically upper respiratory and pneumonia), grade 1/2 acute kidney injury in 45%, and grade 1/2 diarrhea in 40%. No treatment-emergent hypertension or heart failure was noted. One patient died on study, from septic shock due to multilobar pneumonia 7 weeks after completion of induction (eight cycles), with a response of very good partial response (VGPR).

**Fig. 2:**
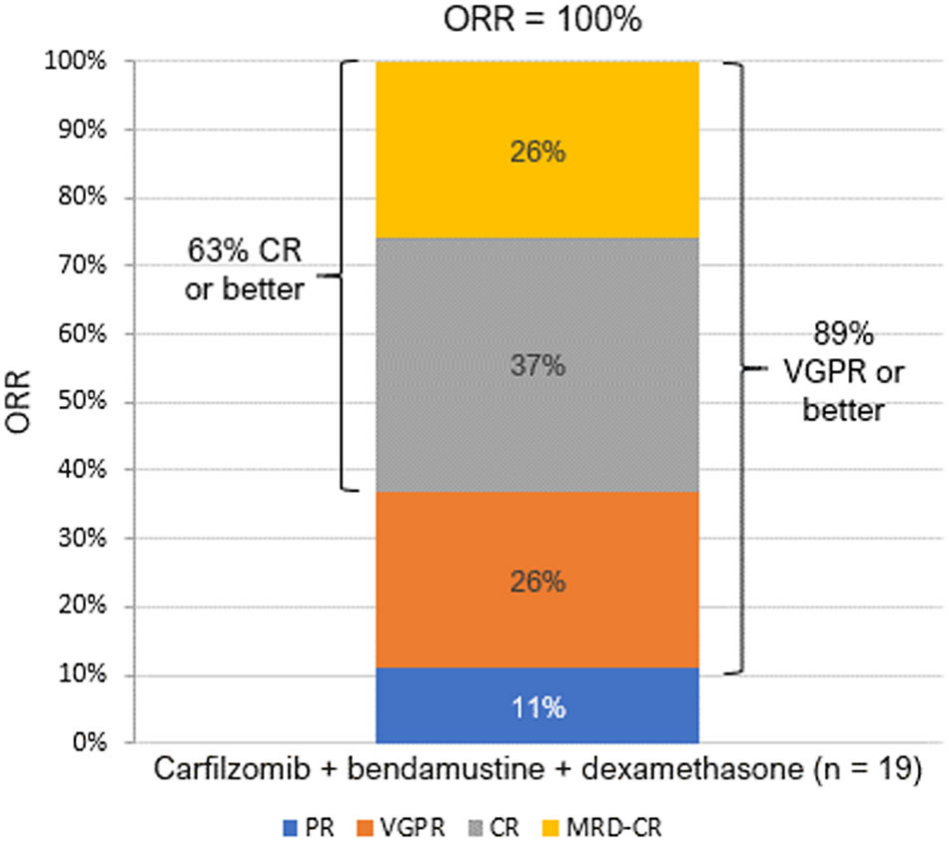
Best response to treatment. CR complete response, MRD minimal residual disease, ORR overall response rate, PR partial response, VGPR very good partial response. All CRs were confirmed by bone marrow aspiration.

CBD was highly effective, with ORR (≥partial response, PR) of 100%. The best responses were 2 (11%) PR, 5 VGPR (26%), and 12 CR (63%, all confirmed) (Fig. 2). Among CRs, four were MRD-positive, five MRD-negative, and three did not have testing. Among PRs, one was treated at dose level 2, then underwent SCT followed by 2 years of carfilzomib maintenance and then observation, and has remained in PR 5 years after diagnosis without relapse; the other was lost to follow-up after completion of induction. Responses were rapid, with a median time to PR of 2.1 months and VGPR of 6.5 months.

With median follow-up of 28 months (range 11–71), two patients have progressed: one with del(17p) at 19 months after diagnosis, and the other with standard cytogenetic risk disease at 56 months. Two patients have died—the patient with del(17p) from refractory myeloma at 37 months after diagnosis, and the other from septic shock as discussed above. Median PFS was 56 months, and median OS has not been reached.

We believe that CBD is a good option for induction in both SCT-eligible and ineligible patients because of several advantages. This regimen does not use an IMID—thereby allowing IMIDs to be saved for use in relapse—but appears to achieve response rates comparable to regimens containing PI and IMID. Though we have not conducted a cost-effectiveness analysis, CBD appears to be less expensive than regimens containing IMIDs. While we gave carfilzomib twice weekly here, once-weekly administration can also be considered^15^, and such a change would yield a regimen not significantly different from PI-IMID regimens in terms of the number of infusion visits, without the worry of patient adherence to oral medications.

The infections we observed (grade 3–4 in 20%) are likely related to the substantial rates of lymphocytopenia and neutropenia with CBD. Grade 1/2 acute kidney injury occurred at a higher rate (45% of patients) than that which has been reported^2,3,5,9^—all events were grade 1, no patient developed tumor lysis, and three of nine patients developed renal injury during their first two cycles of treatment, possibly related to yet-uncontrolled myeloma. Otherwise, the renal injury is possibly related to carfilzomib (though all patients received hydration by protocol). Another consideration is duration of induction—four patients were unable to complete eight cycles of induction due to cumulative toxicity. In future uses of this regimen, shortening the duration to six cycles may be reasonable, particularly if the patient appears to have attained CR.

The limitations of our study include a small sample size, failure to complete planned accrual, and lack of high cytogenetic risk patients. Despite these concerns, we believe that our data provide preliminary evidence that CBD is a tolerable induction regimen that has promising anti-myeloma activity. The high rate of CRs we noted in patients with standard risk disease is particularly encouraging.
